# High-speed fluorescence lifetime imaging microscopy: techniques, applications, and prospects

**DOI:** 10.1117/1.BIOS.2.3.030901

**Published:** 2025-06-05

**Authors:** Hiroshi Kanno, Fan Li, Jongchan Park, Hidenori Endo, Kuniyasu Niizuma, Liang Gao, Keisuke Goda

**Affiliations:** a University of Tokyo, School of Science, Department of Chemistry, Tokyo, Japan; b Tohoku University Graduate School of Medicine, Department of Translational Neuroscience, Miyagi, Japan; c University of California, School of Engineering, Department of Bioengineering, Los Angeles, USA; d Tohoku University Graduate School of Medicine, Department of Neurosurgery, Miyagi, Japan; e Tohoku University, Graduate School of Biomedical Engineering, Department of Neurosurgical Engineering, Miyagi, Japan; f University of California, California NanoSystems Institute, Los Angeles, USA; g Wuhan University, Institute of Technological Sciences, Hubei, China; h Tohoku University, International Center for Synchrotron Radiation Innovation Smart (SRIS), Miyagi, Japan

**Keywords:** fluorescence lifetime, high-speed imaging, fluorescence lifetime imaging microscopy

## Abstract

**Significance:**

Fluorescence lifetime imaging microscopy (FLIM) has been gaining increasing attention due to its capability to provide robust, accurate, and quantitative measurements compared with conventional intensity-based fluorescence microscopy. However, the slow imaging speed of FLIM has long been a major limitation for expanding its applications in biology and medicine.

**Aim:**

We aim to discuss recent advancements in FLIM to enhance imaging speed and explore its biomedical applications and future prospects.

**Approach:**

We discuss high-speed FLIM techniques by categorizing them into time-domain and frequency-domain approaches, as well as wide-field and beam-scanning imaging schemes, with a focus on their combinations.

**Results:**

Recent advances in high-speed FLIM have been primarily driven by innovations in fluorescence detection schemes for wide-field imaging, as well as fluorescence excitation strategies for beam-scanning imaging. By enhancing FLIM imaging speed to levels comparable with intensity-based fluorescence microscopy, the observation of fast cellular dynamics, such as neural spiking, and large-scale image-based analysis of heterogeneous cells have become feasible. In addition, promising directions for the future development of high-speed FLIM include further utilization of fit-free analysis, implementation of video-rate 3D FLIM, and realization of FLIM-based cell sorting.

**Conclusions:**

The development of high-speed FLIM promises new opportunities in cell biology, biophysics, and neurology.

Statement of Discovery Recent advances in high-speed fluorescence lifetime imaging microscopy (FLIM) enable rapid, quantitative imaging of fast cellular dynamics, such as neural spiking and large-scale analysis of heterogeneous cells. The review highlights advancements that expand high-speed FLIM applications in cell biology, biophysics, and neurology, paving the way for real-time 3D imaging and FLIM-based cell sorting.

## 1 Introduction

Fluorescence lifetime imaging microscopy (FLIM) has been gaining increasing attention due to its capability to provide robust, accurate, and quantitative measurements, surpassing conventional intensity-based fluorescence microscopy.^1^^–^^3^ Unlike fluorescence intensity, which is influenced by factors such as fluorophore concentration, photobleaching, and excitation laser intensity, fluorescence lifetime remains largely independent of these variables, ensuring precise and reproducible imaging under varying experimental conditions.^1^^,^^4^ Moreover, fluorescence lifetime is highly sensitive to the microenvironment of fluorescent molecules, particularly when their structural properties are inherently dynamic or chemically engineered to respond to specific stimuli.^1^^,^^4^ For example, FliptR,^5^ commercially known as Flipper-TR,^6^ undergoes conformational changes in response to cell membrane tension, allowing the measurement of this parameter through changes in fluorescence lifetime. This unique capability enables FLIM to correlate fluorescence lifetime with key physiological factors such as pH,^7^^,^^8^ temperature,^9^^,^^10^ viscosity,^11^^–^^13^ ion concentrations,^14^^,^^15^ and metabolic states,^16^^–^^18^ facilitating in-depth studies of both intracellular and extracellular environments with high spatial resolution. Furthermore, fluorescence lifetime can serve as an additional dimension for distinguishing fluorescent molecules with overlapping emission spectra, thus enabling advanced multi-color imaging.^19^^–^^21^ These features make FLIM a powerful and reliable imaging tool across a wide range of fields, including cell biology, biophysics, and neurology.^22^

Despite these advantages, the imaging speed of FLIM (i.e., frame rate) has long been a critical bottleneck, limiting its applicability to studying fast biological processes and statistically analyzing large sample sets. This limitation stems from the inherent complexity and time-consuming nature of fluorescence lifetime measurements, which, unlike fluorescence intensity measurements, require time-resolved detection of fluorescence intensity decay following pulsed excitation. For instance, the time-correlated single-photon counting (TCSPC) technique, widely regarded as the gold standard for fluorescence lifetime measurements, requires thousands of short excitation pulses to construct a single fluorescence decay curve for a single spot.^3^^,^^22^^,^^23^ This is because the excitation pulse intensity must be kept sufficiently low to maintain a fluorescence photon detection probability of ideally less than 0.1 per pulse,^22^^,^^23^ as single-photon avalanche diodes (SPADs), commonly used in TCSPC, can only detect the first arriving fluorescence photon during each excitation cycle and ignore subsequent photons due to their dead time.^24^^,^^25^ These technical requirements collectively result in slower data acquisition compared with fluorescence intensity measurements, thereby posing challenges for real-time imaging and high-throughput applications.

This review provides a comprehensive overview of recent advances in high-speed FLIM, emphasizing the principles and techniques that have enabled rapid imaging and their applications in biological and medical research. Specifically, it covers progress in both time-domain and frequency-domain FLIM, including wide-field and beam-scanning approaches. In addition, we discuss how these advancements have expanded FLIM’s ability to investigate complex biological systems with greater precision and efficiency. Finally, we highlight the remaining challenges and emerging opportunities in this evolving field, offering insights into future directions for improving FLIM’s speed, sensitivity, and applicability in cutting-edge research.

## 2 Principles of High-Speed FLIM

**Fig. 1:**
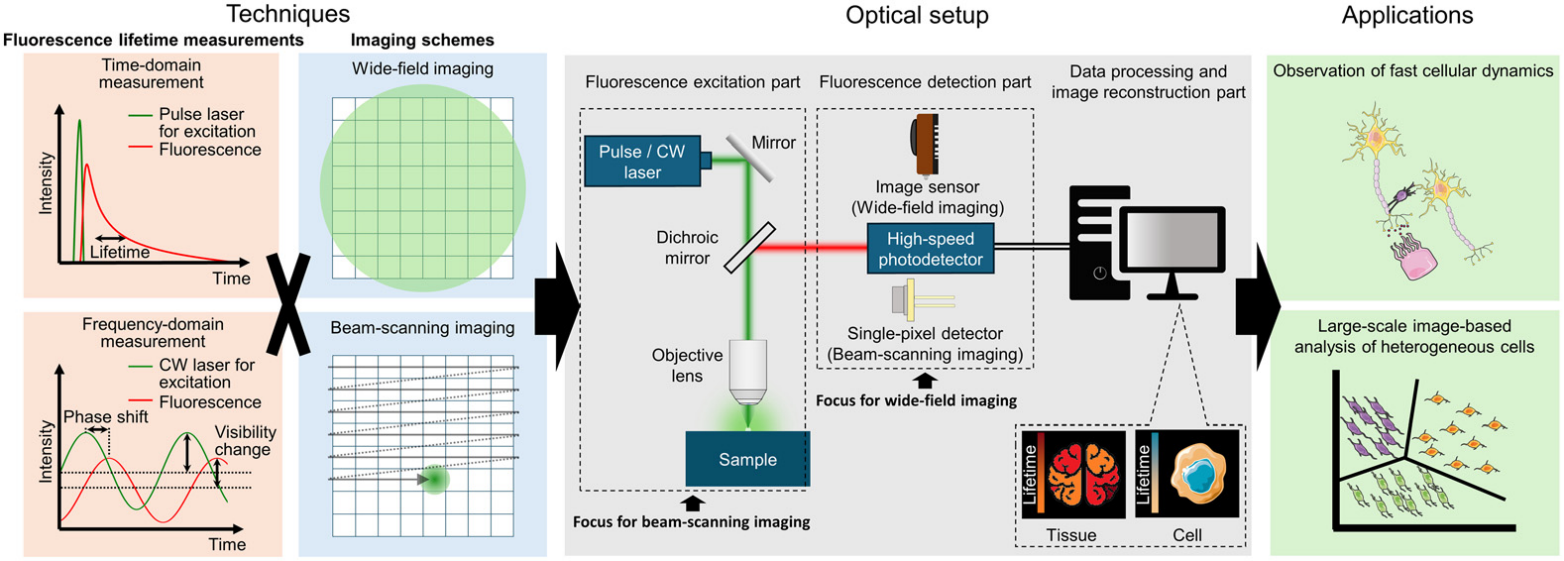
Overview of high-speed FLIM. FLIM techniques are classified by their fluorescence lifetime measurements (time-domain or frequency-domain measurements) and imaging schemes (wide-field or beam-scanning schemes). The optical setup is designed accordingly: time-domain FLIM employs pulse lasers, whereas frequency-domain FLIM typically uses continuous-wave (CW) lasers; wide-field FLIM utilizes image sensors, whereas beam-scanning FLIM generally relies on single-pixel detectors. In wide-field FLIM, the focus is on how to achieve temporal resolution of fluorescence signals, whereas in beam-scanning FLIM, the emphasis is mainly on how to achieve high-speed beam scanning.

To overcome the speed limitations of conventional FLIM, advancements in fluorescence excitation and/or detection techniques are essential. Traditional FLIM is typically restricted to a few frames per second at most, but innovations in these areas have enabled significantly faster image acquisition rates, reaching video rates or beyond. FLIM techniques can be broadly classified into four main categories based on their combination of time-domain or frequency-domain approaches with different imaging geometries: (1) time-domain wide-field FLIM, (2) time-domain beam-scanning FLIM, (3) frequency-domain wide-field FLIM, and (4) frequency-domain beam-scanning FLIM (Fig. 1). In this section, we introduce and discuss representative high-speed FLIM techniques in each category, highlighting their key features and the innovations that facilitate high-speed imaging applications (Table 1). It should be noted that the FLIM frame rate depends on the targeted fluorescence lifetime, and thus, we focus here on FLIM targeting typical fluorescence lifetimes ranging from sub-nanoseconds to several nanoseconds.

**Table 1:** Comparison of high-speed FLIM techniques.

Scheme^a^	Light source	Detector type	Key feature^b^	Demonstrated fps^c^	Potential drawback^d^	Ref.
TD, WF	Ti: sapphire laser, 700 to 1000 nm, <2 ps, 82 MHz	CCD image sensor	Time gating and time delay technique via an additional optical path.	∼100 fps	Limitations in image quality due to an image intensifier. Low fluorescence collection efficiency.	^26^
TD, WF	Supercontinuum laser, 450 to 500 nm, 100 ps, 39 MHz	sCMOS image sensor	Fast, wide-field time gating using a Pockels cell.	∼1000 fps	Relatively slow and potentially spatially nonuniform time gating by a Pockels cell.	^27^
TD, WF	Picosecond pulsed laser, 515 nm	Streak camera (CMOS image sensor)	Fast fluorescence decay imaging via compressed sensing with a streak camera.	∼100 fps	Computationally intensive image reconstruction. Reduced SNR due to image compression.	^28^
TD, BS	FDML-MOPA laser, 1060 nm, 65 ps, 88 MHz	Hybrid photodetector	Fast, serial TPF excitation by spectrally encoded pulse train.	∼1000 fps	Spatial binning required to compensate for low photon counts, at the expense of lifetime accuracy.	^29^
TD, BS	Ti: sapphire laser, 750 to 800 nm, 100 fs, 80 MHz	PMT	Real-time phasor by GPU-accelerated processing using TPF.	∼25 fps	Limited imaging speed by 2D beam scanning.	^30^
FD, WF	CW laser, 442 nm, 1 W	CCD image sensor	Wide-field fluorescence phase estimation with a phase-sensitive image sensor.	∼20 fps	Limited imaging speed due to sensor switching rate. Relatively high readout noise of the sensor.	^31^
FD, BS	CW laser, 488 nm, 2 W	APD	Parallel excitation by dual frequency-encoded intensity-modulated beam arrays.	∼10,000 fps	Increased shot noise due to parallel fluorescence excitation. Relatively low APD gain.	^32^
FD, BS	Ti: sapphire laser, 710 to 990 nm, 100 fs, 80 MHz	PMT	Fast, cost-effective signal processing for TPF lifetime calculation.	∼8 fps (assuming 100 × 100 pixels)	Limited imaging speed by 2D beam scanning.	^33^

a TD: time domain, FD: frequency domain, WF: wide field, BS: beam scanning

b TPF: two-photon fluorescence

c fps: frames per second

d QE: quantum efficiency, SNR: signal-to-noise ratio

### 2.1 Time-domain Wide-field FLIM

Time-domain wide-field FLIM is a technique to simultaneously estimate fluorescence lifetimes across the entire sample from their fluorescence intensity decays using pulse excitation. It primarily relies on a time-gating scheme, which divides the fluorescence signal into multiple time intervals and sequentially measures the fluorescence intensity within each interval, thereby reconstructing or estimating the original fluorescence decay curve for the entire image.^34^ This approach is necessary for time-domain wide-field FLIM because image sensors, such as charge-coupled device (CCD) or complementary metal oxide semiconductor (CMOS) image sensors, lack intrinsic temporal resolution in their pixels to record fluorescence intensity decay curves after pulse excitation. To compensate for this limitation, additional mechanisms such as image intensifiers are employed to apply time gates and capture fluorescence signals at different time points. However, this time-gating approach typically requires multiple excitation-detection cycles to obtain fluorescence intensity images across all time intervals, which inherently limits the imaging speed.

**Fig. 2:**
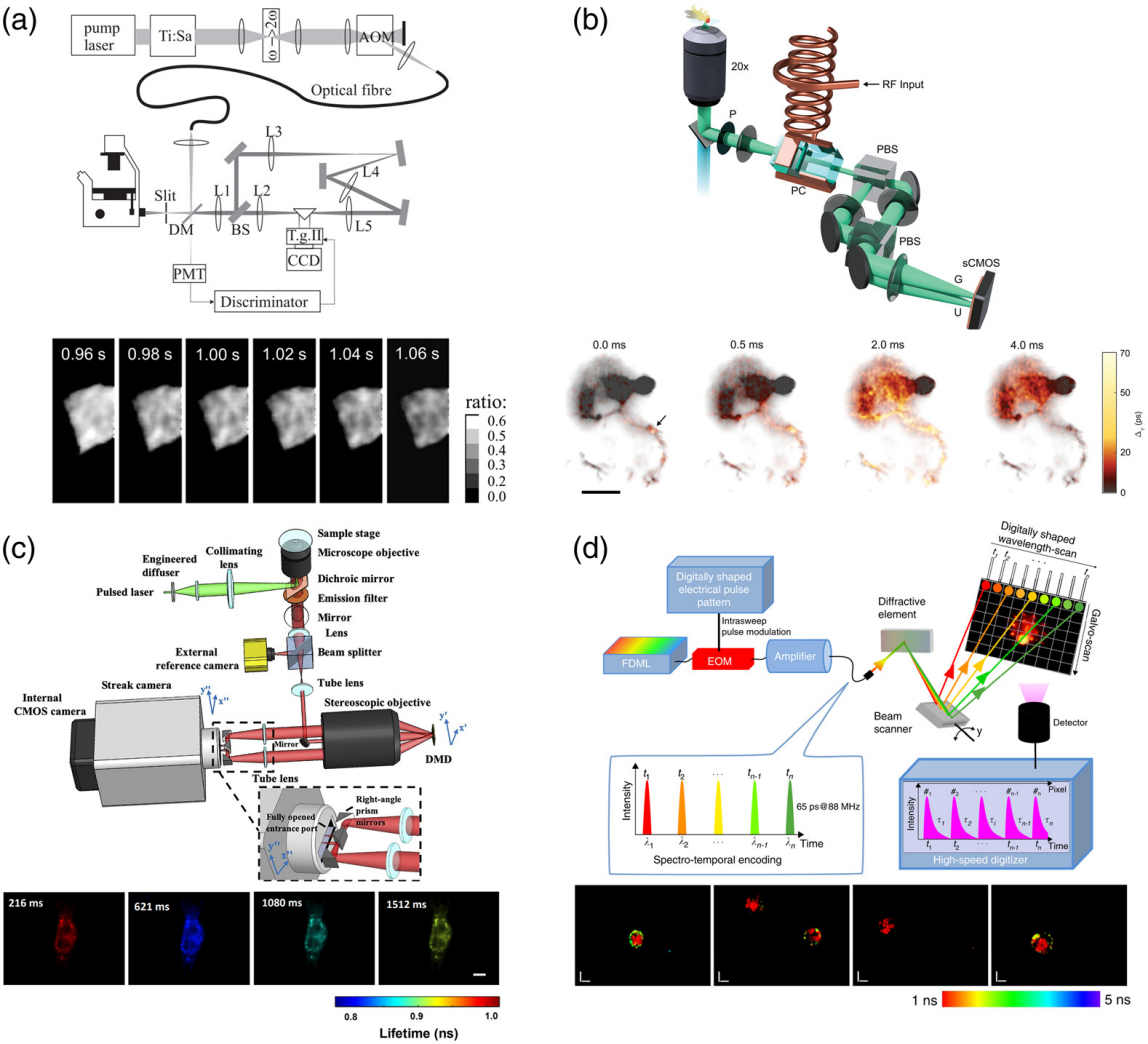
High-speed time-domain FLIM. (a) Wide-field FLIM using a time-gating scheme with an image intensifier. The beam splitter (BS) and optical path difference introduced by it generate a pair of fluorescence intensity images in different time windows. Representative fluorescence intensity ratio images of a rat neonatal myocyte stained with Oregon green bapta-1 are also presented. Each image size is 24  μm (horizontal)×63  μm (vertical). Adapted from Ref. 26 with permission from IOP Publishing. (b) Wide-field FLIM using a time-gating scheme with a Pockels cell. P: polarizer, PC: Pockels cell. Representative fluorescence lifetime images of a neuron in a *Drosophila melanogaster* fly expressing pAce^37^ are also presented. Scale bar: 25  μm. Adapted from Ref. 27. Reused with permission from AAAS, copyright 2023. (c) Wide-field FLIM using a streak camera. Representative fluorescence lifetime images of a cardiomyocyte expressing MacQ-mOrange2^38^ are also presented. Scale bar: 10  μm. Adapted from Ref. 28 with permission from PNAS. (d) Beam-scanning FLIM using a spectrally encoded pulse train for serial fluorescence excitation. EOM: electro-optic modulator. Representative fluorescence lifetime images of *Euglena gracilis* cells stained with Nile Red are also presented. Scale bar: 10  μm. Adapted from Ref. 29, licensed under CC BY 4.0.

To address this limitation, several strategies have been proposed. One notable approach involves constructing fluorescence lifetime images from the ratio of intensity images acquired during the early and late halves of the fluorescence decay.^35^^,^^36^ As a practical implementation of this approach, Agronskaia et al. demonstrated a technique that enables the simultaneous acquisition of fluorescence intensity image pairs.^26^ In this technique, the fluorescence signal from a sample is split into two optical paths using a beam splitter, with a temporal delay introduced in one path by inserting a 1.515 m relay lens, corresponding to a ∼5  ns time delay [Fig. 2(a)]. The fluorescence image pairs, captured at different time points after emission, are then imaged on a single CCD following time gating by an image intensifier and subsequently used for fluorescence lifetime calculation. This design allows for frame rates of up to 100 frames per second. Despite this improvement in imaging speed, the technique suffers from low fluorescence collection efficiency, as the beam splitter used to generate the fluorescence intensity image pairs discards a significant portion of the emitted fluorescence. This limitation becomes particularly critical in high-speed imaging scenarios, where the photon collection time per image is already constrained, potentially degrading the precision of fluorescence lifetime calculations. In addition, the photocathode and microchannel plate inside the image intensifier may degrade image quality.^39^

An alternative approach to obtaining fluorescence intensity ratios while minimizing fluorescence loss is the utilization of electro-optic effects. Bowman et al. achieved time-gated fluorescence detection with minimal fluorescence loss by employing a custom-made Pockels cell to rapidly modulate the polarization of the emitted fluorescence.^27^^,^^40^^,^^41^ Figure 2(b) illustrates a schematic of their technique, in which the Pockels cell is placed between a polarizer and polarizing beam splitter (PBS) and driven by a sinusoidal radio-frequency signal synchronized with half the pulse repetition rate. The 90-deg polarization rotation of fluorescence induced by the Pockels cell allows the PBS to separate the early and late halves of the fluorescence decay into distinct optical paths, enabling both fluorescence intensity images to be captured on a single CMOS sensor. By utilizing the fluorescence polarization component that is currently lost at the polarizer, fluorescence loss due to time gating can be avoided.^41^ This configuration maximizes fluorescence signal utilization for fluorescence lifetime calculation, as no fluorescence signal is discarded, thus improving both speed and sensitivity. Using this approach, they achieved kilohertz frame-rate *in vivo* FLIM of *Drosophila*.^27^

Streak cameras, known for their superior temporal resolution in single-shot measurements, have been explored as an effective tool for temporally resolving fluorescence intensity. However, conventional streak cameras function as one-dimensional imaging devices. They temporarily disperse an incident line image using an optoelectronic streaking unit and map the result onto a 2D imaging camera. Therefore, capturing a full 2D FLIM image typically requires extensive scanning across the field of view (FOV), significantly limiting imaging speed. To overcome this limitation, Ma et al. introduced a single-shot high-speed FLIM technique by leveraging the compressed sensing (CS) principle [Fig. 2(c)].^28^ Their key innovation was integrating a streak camera with a spatial encoding mask, a concept originally devised for achieving ultrafast imaging at a frame rate of 0.1 THz,^42^ as explained in a tutorial article.^43^ In their approach, a 2D spatial encoding mask modulates the fluorescence image, imprinting a random binary pattern onto it. This modulated image is then detected by the streak camera. A CS algorithm reconstructs the fluorescence lifetime images from this single, spatiotemporally mixed dataset, enabling single-shot measurements. As a result, they achieved high-speed FLIM at 100 frames per second. Although the photocathode and microchannel plate in the streak camera may degrade image quality, similar to image intensifiers,^39^ this issue can be mitigated by utilizing both fluorescence images masked with the complementary on- and off-state binary patterns of the digital micromirror device, as well as a fluorescence image obtained by a reference camera.

### 2.2 Time-domain Beam-scanning FLIM

Time-domain beam-scanning FLIM is a technique that performs pulse excitation and fluorescence detection on a pixel-by-pixel basis, directly recording fluorescence intensity decay curves for each pixel. Unlike image sensors, single-pixel photodetectors, such as photomultiplier tubes (PMTs) and avalanche photodiodes (APDs), inherently possess temporal resolution, making the use of time-gating schemes unnecessary in this technique. This capability simplifies the detection mechanisms compared with wide-field FLIM. However, the mechanical inertia associated with beam scanning imposes significant challenges for high-speed imaging, as it slows down the process and limits overall frame rates. Therefore, optimizing the fluorescence excitation schemes is crucial for enhancing the imaging speed of FLIM.

One effective strategy is to circumvent the time-intensive 2D scanning process in beam-scanning fluorescence lifetime measurements. Karpf et al. demonstrated a technique employing a Fourier-domain mode-locked (FDML) laser^44^^,^^45^ in combination with an electro-optic modulator to generate a spectrally encoded pulse train for rapid and sequential two-photon fluorescence excitation [Fig. 2(d)].^29^ This approach builds on the concept of encoding spatial information into a pulse spectrum, which has been extensively explored over the past two decades—achieving ultrafast imaging with frame rates in the megahertz range or line acquisition rates from tens to a hundred megahertz—but has been predominantly applied to bright-field and quantitative phase imaging.^46^^–^^52^ In this approach, the spectrally encoded pulse train is spatially distributed by a diffractive optical element, such as a diffraction grating, establishing a one-to-one correspondence between light wavelength and spatial position in one dimension. In addition, the temporally spaced pulses, with intervals of ∼12  ns, are sequentially detected and temporally resolved by a single-pixel photodetector with high temporal resolution. As a result, high-speed two-photon fluorescence lifetime imaging at kilohertz frame rates becomes possible by performing the fast-axis scan without mechanical scanning, bypassing the limitations of conventional 2D scanning FLIM. This technique enhances imaging speed and offers a promising solution for high-speed FLIM applications.

In the context of video-rate two-photon excitation FLIM, the combination of fast raster scanning and direct temporal measurement of fluorescence pulses remains a practical and effective approach.^53^ For instance, Bower et al. demonstrated the imaging of nicotinamide adenine dinucleotide (NADH) and the tracking of transient metabolic dynamics at a video-rate level by performing deconvolution of the instrument response function followed by curve fitting of the two-photon fluorescence signal.^54^^–^^56^ Using a similar optical setup, Sorrells et al. developed a GPU-accelerated real-time phasor analysis technique^30^ (see Sec. 4.2 for phasor analysis) and a computational photon-counting algorithm,^57^ achieving photon counting at twice the laser repetition rate^57^ and real-time lifetime calculation. As noted in these studies, video-rate FLIM of NADH autofluorescence often requires frame averaging to improve the accuracy of lifetime measurements, primarily due to the inherently low two-photon cross-section and quantum yield of endogenous fluorophores.^58^^,^^59^ By contrast, video-rate FLIM using exogenous dyes has been demonstrated without frame averaging. For example, Rhodamine B uptake by cells was successfully imaged at 25 frames per second with a reasonable signal-to-noise ratio.^30^

A few commercial video-rate FLIM systems based on the time-domain beam-scanning technique are also available. Specifically, the SP8 FALCON from Leica Microsystems Inc. can operate as a video-rate FLIM system^60^ and has been applied in various biological studies, such as intracellular calcium imaging^61^ and subcellular pH quantification.^7^ It digitizes excitation and fluorescence pulses at 10 GHz and calculates their arrival time differences using a field-programmable gate array (FPGA), enabling direct fluorescence lifetime estimation with reduced jitter. In addition, the use of multiple detectors supports high photon flux and, with a pile-up^22^^–^^25^ suppression filter, allows for accurate, high-speed FLIM. A similar concept, using multiple detectors to simultaneously capture photons from distinct fluorescence wavelength ranges, was also adopted to increase photon flux in a clinical demonstration of FLIM.^62^ Furthermore, another commercial system, rapidFLIM from PicoQuant GmbH, also achieves 20 frames per second within a limited FOV (64×64  pixels) by minimizing the dead time in its detection system.^63^

### 2.3 Frequency-domain wide-field FLIM

Frequency-domain wide-field FLIM is typically performed by exciting an entire fluorescent sample with continuous-wave (CW) light whose intensity is sinusoidally modulated.^1^^,^^4^ The fluorescence lifetime is calculated by measuring the phase shifts and/or modulation depth reduction (i.e., visibility change) between the excitation and fluorescence intensity signals.^4^ Because conventional image sensors lack the required temporal resolution, additional mechanisms are employed for sensing temporal phases of fluorescence intensity signals, similar to those used in time-domain wide-field FLIM approaches. A common approach involves an image intensifier whose gain modulation is synchronized with the excitation intensity modulation. By acquiring multiple fluorescence intensity images (at least three,^64^ but typically twelve images^31^) while varying the initial phase of the gain modulation, both the phase and modulation depth of the fluorescence signal can be extracted. However, acquiring multiple images for a single fluorescence lifetime image is time-consuming, thereby limiting the overall acquisition speed.

**Fig. 3:**
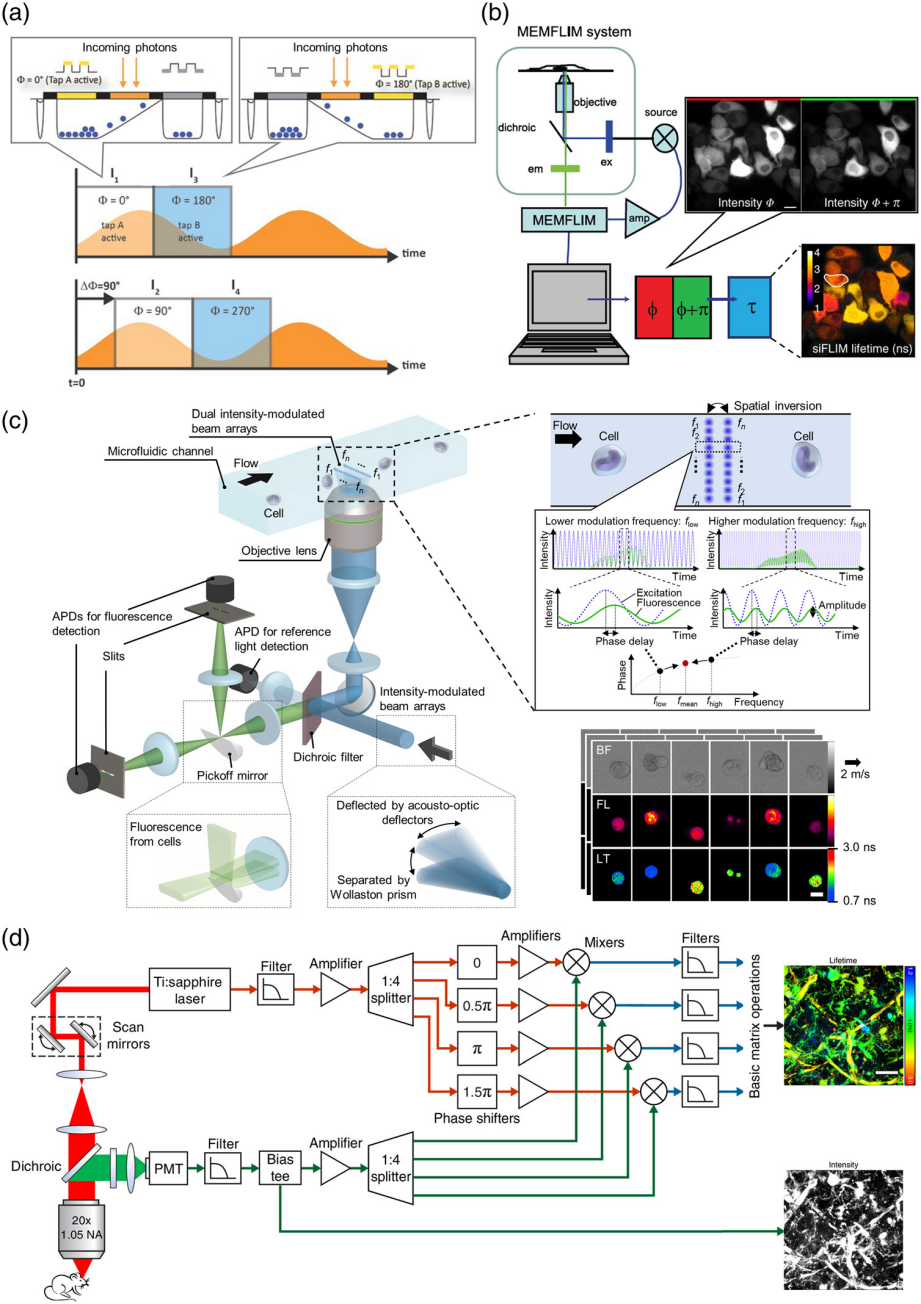
High-speed frequency-domain FLIM. (a) Principle of a phase-sensitive image sensor. Adapted from Ref. 67, with permission from the author and SPIE. (b) Wide-field FLIM with a phase-sensitive image sensor. MEMFLIM stands for a modulated-FLIM camera. A representative fluorescence intensity image pair with a π-phase shift and the resulting fluorescence lifetime image of HeLa cells expressing a cAMP sensor^68^ are also presented. Scale bar: 10  μm. Adapted from Ref. 31, with permission from Springer Nature. (c) Beam-scanning FLIM using dual intensity-modulated beam arrays. Representative bright-field (BF), fluorescence intensity (FL), and fluorescence lifetime (LT) images of Jurkat cells stained with SYTO16 are also presented. Scale bar: 10  μm. Adapted from Ref. 32, licensed under CC BY 4.0. (d) Instant FLIM system. Maximized z-projection images of fluorescence lifetime and intensity from a fixed Cx3cr1-GFP/+ mouse brain are also presented. Scale bar: 20  μm. Adapted from Ref. 33, under the Optica Publishing Group Open Access Publishing Agreement (OAPA).

To address this limitation, a phase-sensitive image sensor was applied to wide-field frequency-domain FLIM.^39^^,^^65^^–^^67^ As illustrated in Fig. 3(a), the image sensor can rapidly switch between two distinct channels that separately integrate incoming photons, with a switching frequency of tens of megahertz.^67^ This enables the estimation of fluorescence modulation amplitudes and phases in a wide-field manner. Raspe et al. demonstrated fast fluorescence lifetime image acquisition by recording a single π-phase-shifted fluorescence intensity image pair [Fig. 3(b)].^31^ By appropriately adjusting the initial phase of the sorting mechanism and integrating fluorescence photons over half of the excitation modulation cycles, a single intensity image pair provides sufficient information to calculate a fluorescence lifetime image. This approach removes the need for sequential phase-shifting steps, which are a major bottleneck in conventional approaches. Moreover, their FLIM technique offers advantages over image intensifier-based systems in terms of photon efficiency, spatial resolution, and resilience to overexposure.^31^ As a result, they achieved video-rate time-lapse FLIM of cultured cells and demonstrated the capability for video-rate, prolonged observation of intracellular fluorescence lifetime changes affected by calcium ion levels. A commercial FLIM camera based on this principle, pco.flim from Excelitas PCO GmbH, is available and has been tested in cellular imaging applications.^69^

### 2.4 Frequency-domain Beam-scanning FLIM

Frequency-domain beam-scanning FLIM is a technique for acquiring fluorescence lifetime images by raster-scanning a sample with a single-spot CW laser or light whose intensity is sinusoidally modulated. Although single-pixel photodetectors provide temporal resolution of fluorescence signals and high-speed readout, the primary challenge for achieving high-speed FLIM lies in overcoming the low signal-to-noise ratio (SNR) caused by short pixel dwell times during fast scanning. Frequency-division-multiplexed (FDM) imaging is an emerging approach to multiplex beam-scanning imaging with CW lasers, thereby achieving high-speed imaging.^70^^–^^77^ In this technique, excitation light modulation frequencies are uniquely assigned to 1D spatial positions, enabling the acquisition of 2D images through 1D scanning and enhancing the image SNR [Fig. 3(c)]. However, when fluorescence signals comprise multiple lifetime components, the measured fluorescence lifetimes in FDM imaging vary with the modulation frequencies.^32^ This frequency dependency causes spatial heterogeneity in fluorescence lifetimes across the FOV, even for the same fluorophores. As a result, previous FDM imaging techniques cannot be directly applied to FLIM.

Kanno et al. recently demonstrated high-speed frequency-domain beam-scanning FLIM by addressing the issue of frequency dependency in FDM imaging.^32^ Specifically, they employed dual-beam arrays in FDM, where one beam array is spatially inverted relative to the other. This design allows each spatial point in the sample to be scanned using paired modulation frequencies [Fig. 3(c)]. The beam arrays are generated by driving two acousto-optic deflectors (AODs) with multi-tone signals and interfering the two sets of deflected beams from them. One of the beam arrays is inverted using a mirror pair and then separated by a Wollaston prism in the direction perpendicular to the beam spot aligning direction. Although emitted intensity-modulated fluorescence signals from each beam array are simultaneously detected by an APD, performing signal processing, including Fourier transformation, on the detected signals can separate and demodulate each beam signal, enabling the calculation of their amplitudes and phases. The obtained phase image pairs are subsequently merged to generate a single fluorescence lifetime image, effectively mitigating the frequency dependency in fluorescence lifetime measurements based on the FDM imaging concept. As a result, they achieved frame rates exceeding 10,000 frames per second, enabling high-throughput FLIM-based flow cytometry.

Video-rate FLIM based on two-photon excitation, as discussed in Sec. 2.2, can also be classified as frequency-domain beam-scanning FLIM due to its detection scheme. Specifically, Zhang et al. developed a system referred to as instant FLIM for *in vivo*, time-lapse 3D (i.e., 4D) FLIM imaging of biological samples using two-photon excitation at video-rate 2D frame rates.^33^ Although it may not be categorized as high-speed FLIM, the system offers cost-effective signal detection and fast lifetime calculation in a manner conceptually similar to analog lock-in detection. As shown in Fig. 3(d), the excitation reference signal is split into four paths with phase shifts of 0, 0.5π, π, and 1.5π, respectively. The detected fluorescence signal is similarly split and mixed with the phase-shifted references. After low-pass filtering, the resulting DC components are measured without the need for high-speed digitizers and used for lifetime calculation. With a pixel dwell time of 12  μs, the system achieves a frame rate of up to 8.3 frames per second for an assumed image size of 100×100  pixels. Further speed improvements are possible through faster beam scanning using resonant or polygon scanners.^33^ The authors demonstrated long-term 4D imaging of zebrafish and mouse brains, showcasing the potential of the developed system for biological studies.

## 3 Applications of high-speed FLIM

High-speed FLIM techniques introduced in the previous section, each employing a distinct imaging scheme, have surpassed the imaging speed limitations of conventional FLIM techniques. These advancements have paved the way for biomedical applications that were previously unattainable. Although research into the potential applications of high-speed FLIM is still in its nascent stages, the results indicate its strong potential for future applications. In this section, we specifically focus on three key applications of high-speed FLIM: the observation of fast neuronal activity, video-rate intracellular temperature monitoring, and fluorescence lifetime imaging flow cytometry.

### 3.1 Observation of Fast Neuronal Activity

**Fig. 4:**
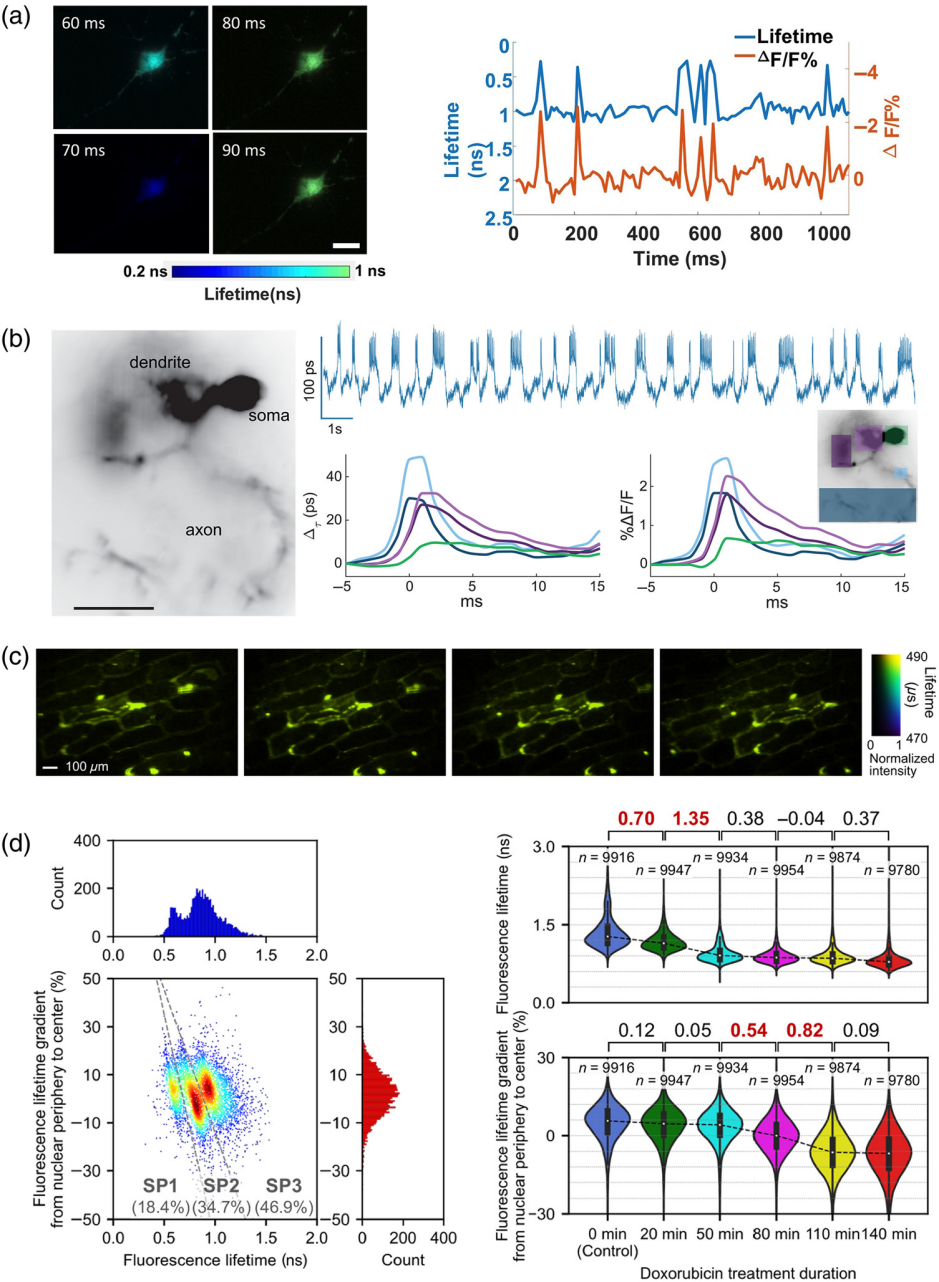
Biomedical applications of high-speed FLIM. (a) Time-lapse observation of neural spiking, averaged across a single neuron (right), and fluorescence lifetime images at representative time points of a hippocampal neuron expressing GEVI (MacQ-mOrange2) (left). Scale bar: 10  μm. Adapted from Ref. 28, with permission from PNAS. (b) Wide-field image of a neuron (left) in a *Drosophila melanogaster* fly, expressing GEVI (pAce), whole-cell lifetime trace (upper right), and average spike shapes in intensity and lifetime from colored regions (bottom right). Scale bar: 25  μm. Adapted from Ref. 27, with permission from AAAS. (c) Photoluminescence lifetime images of moving onion epidermis cells labeled with UCNPs. Adapted from Ref. 10, licensed under CC BY 4.0. (d) Large-scale image-based analysis with high-speed FLIM. The left scatter plot shows cell distribution within rat glioma stained with SYTO16, whereas the right violin plots show distributions of Jurkat cells treated with doxorubicin for different durations. The fluorescence lifetime gradient from the nuclear periphery to the center is a parameter that can be extracted from fluorescence lifetime imaging flow cytometry. SP: subpopulation. Values on top of each violin plot are for effect sizes (Cohen’s d), indicating the magnitude of the difference between populations. Adapted from Ref. 32, licensed under CC BY 4.0.

Neural spiking, one of the fastest transient events in biology, refers to the rapid electrical signals generated by neurons on a millisecond timescale. This neuronal activity has traditionally been studied using calcium indicators to measure fluorescence intensity. However, these indicators provide an indirect and slower representation of underlying electrical current changes.^38^^,^^78^^,^^79^ Genetically encoded voltage indicators (GEVIs) utilizing Förster resonance energy transfer (FRET)^80^ offer a higher-speed alternative for capturing action potentials.^37^^,^^38^^,^^81^ However, their readout, based on the ratio of relative intensity changes, is highly susceptible to noise factors such as laser fluctuations, photobleaching, and background autofluorescence. By contrast, fluorescence lifetime is unaffected by these artifacts and offers a quantitative measure of fast neuronal dynamics.^82^ Ma et al. demonstrated time-lapse imaging of neural spiking in cultured neurons with FRET-opsin-based GEVI, MacQ-mOrange2 [Fig. 4(a)].^28^ In this sensor, the bright fluorescent protein mOrange2 acts as the FRET donor, whereas the voltage-sensitive opsin Mac, located in close proximity, serves as the FRET acceptor.^38^ Upon neuronal depolarization, the opsin may change its conformation or photophysical properties, enhancing energy transfer from the donor to the acceptor.^38^ This leads to a reduction in both fluorescence intensity and lifetime. By employing single-shot time-domain wide-field FLIM, they measured the fluorescence lifetime of the FRET sensor at 100 Hz, enabling spatially resolved detection of neuronal dynamics. As another example, Bowman et al. demonstrated *in vivo* imaging of action potentials in a *Drosophila melanogaster* fly expressing GEVI, pAce,^37^ composed of the fluorescent protein mNeonGreen (FRET donor) and a voltage-sensitive opsin (FRET acceptor), using an electro-optic-based FLIM system operating at kilohertz frame rates [Fig. 4(b)].^27^ They successfully captured fluorescence lifetime changes on the order of ∼100  ps or smaller, enabling the detection of neuronal action potentials. It should be noted that while both studies reported that the fluorescence lifetime changes are linearly proportional to the fluorescence intensity changes, the observed lifetime changes in Fig. 4(a) are considerably larger than in Fig. 4(b). Although the origin of this discrepancy remains unclear—particularly because the two experiments used different FRET-based voltage sensors—it might reflect complex photophysical or experimental factors that affect lifetime measurement, including the ratio between interacting and non-interacting proteins, the spectral cross-section of donors and acceptors, and the calculating methods for estimating lifetimes. Notably, Ma et al. captured the full fluorescence decay and applied exponential curve fitting, whereas Bowman et al. used only two time bins along the decay and employed a ratiometric approach.

### 3.2 Video-rate Intracellular Temperature Monitoring

Temperature is a crucial factor in regulating various cellular processes, including gene expression and metabolic activity.^83^^,^^84^ Conventional methods for monitoring temperature involve thermography and thermocouples, which suffer from low and no spatial resolution, respectively.^9^^,^^85^ To overcome this limitation, FLIM provides a promising solution, offering high spatial resolution for temperature imaging using temperature-sensitive fluorescent probes. The remaining challenge, however, is temporal resolution to capture rapid temperature changes within cells. In this context, Liu et al. demonstrated video-rate intracellular temperature monitoring using up-conversion nanoparticles (UCNPs) as luminescent thermometers.^10^ Although the photoluminescence lifetime of these nanoparticles ranges from a few hundred microseconds, which is significantly longer than typical fluorescence lifetimes, they used the CS concept and successfully acquired lifetime-based temperature images at 20 Hz [Fig. 4(c)]. Expanding the use of video-rate intracellular temperature monitoring may help address an important unresolved problem in this field: the discrepancy between theoretical predictions and experimentally observed intracellular temperature variations.^86^ Moreover, it may contribute to evaluating the efficacy of chemotherapy and photothermal therapy *in vivo*.^87^^–^^89^

### 3.3 Fluorescence Lifetime Imaging Flow Cytometry

The imaging capability of high-speed FLIM enables large-scale, single-cell image–based profiling of heterogeneous cell populations (i.e., imaging flow cytometry). Because tissues and lesions, such as tumors and blood, typically consist of diverse cell types with various phenotypes and states, a comprehensive and statistically significant analysis of these cells is crucial for gaining deeper insights into complex biological systems.^90^^–^^92^ To address this need, imaging flow cytometry has emerged as a promising tool for cell analysis and has been widely applied across various fields, ranging from basic science to clinical applications.^93^ It provides detailed image-based information on target cells, offering significant advantages over traditional flow cytometry.^94^^,^^95^ However, conventional imaging flow cytometry predominantly relies on fluorescence intensity imaging, making it susceptible to unwanted fluctuations in fluorescence signal intensity and limiting its quantitative accuracy. Although fluorescence-lifetime-based imaging flow cytometry has the potential to overcome these limitations, the slow image acquisition speeds of conventional FLIM techniques have hindered their direct integration into flow cytometric systems. In this context, Karpf et al. and Ma et al. attempted to incorporate their high-speed FLIM techniques into flow cytometry and flow particle analysis, respectively.^28^^,^^29^ Although they successfully demonstrated fluorescence lifetime imaging of flowing cells or particles, the flow speeds remained ≤0.2  m/s, which is insufficient for practical flow cytometry applications.^96^ Moreover, large-scale fluorescence lifetime image dataset analysis was not achieved.

Recently, Kanno et al. developed a high-throughput fluorescence lifetime imaging flow cytometer and demonstrated large-scale fluorescence lifetime image-based analysis of biomedical samples.^32^ Using this system, they experimentally demonstrated event rates exceeding 10,000  cells/s with polymer beads and Jurkat cells.^32^^,^^97^ Furthermore, they validated the system with fluorescence-lifetime-encoded beads^98^ and *Euglena gracilis* cells stained with SYTO16, successfully distinguishing inter-object and intra-object components based on their fluorescence lifetimes—an outcome not achievable with fluorescence intensity-based techniques. Beyond technical validation, they demonstrated the biomedical utility of FLIM flow cytometry. Specifically, they analyzed cellular heterogeneity in rat glioma, including highly aggressive subtypes,^99^ and investigated the drug responses of cancer cells^100^ through large-scale fluorescence lifetime image analysis. As a result, they successfully detected lifetime-based subpopulations within the rat glioma and drug-induced temporal intranuclear dynamics in cancer cells [Fig. 4(d)]. These findings highlight the potential of FLIM flow cytometry as a powerful tool for advancing biomedical research.

## 4 Future Prospects of High-speed FLIM

In this section, we explore the future prospects of high-speed FLIM, highlighting recent advancements such as SPAD arrays and fit-free analysis techniques that provide rapid and intuitive interpretation of FLIM images. In addition, we discuss key development directions, including 3D imaging, spectrally resolved imaging, and the integration of FLIM with intelligent image-activated cell sorting.

### 4.1 SPAD Arrays for High-speed TCSPC

**Fig. 5:**
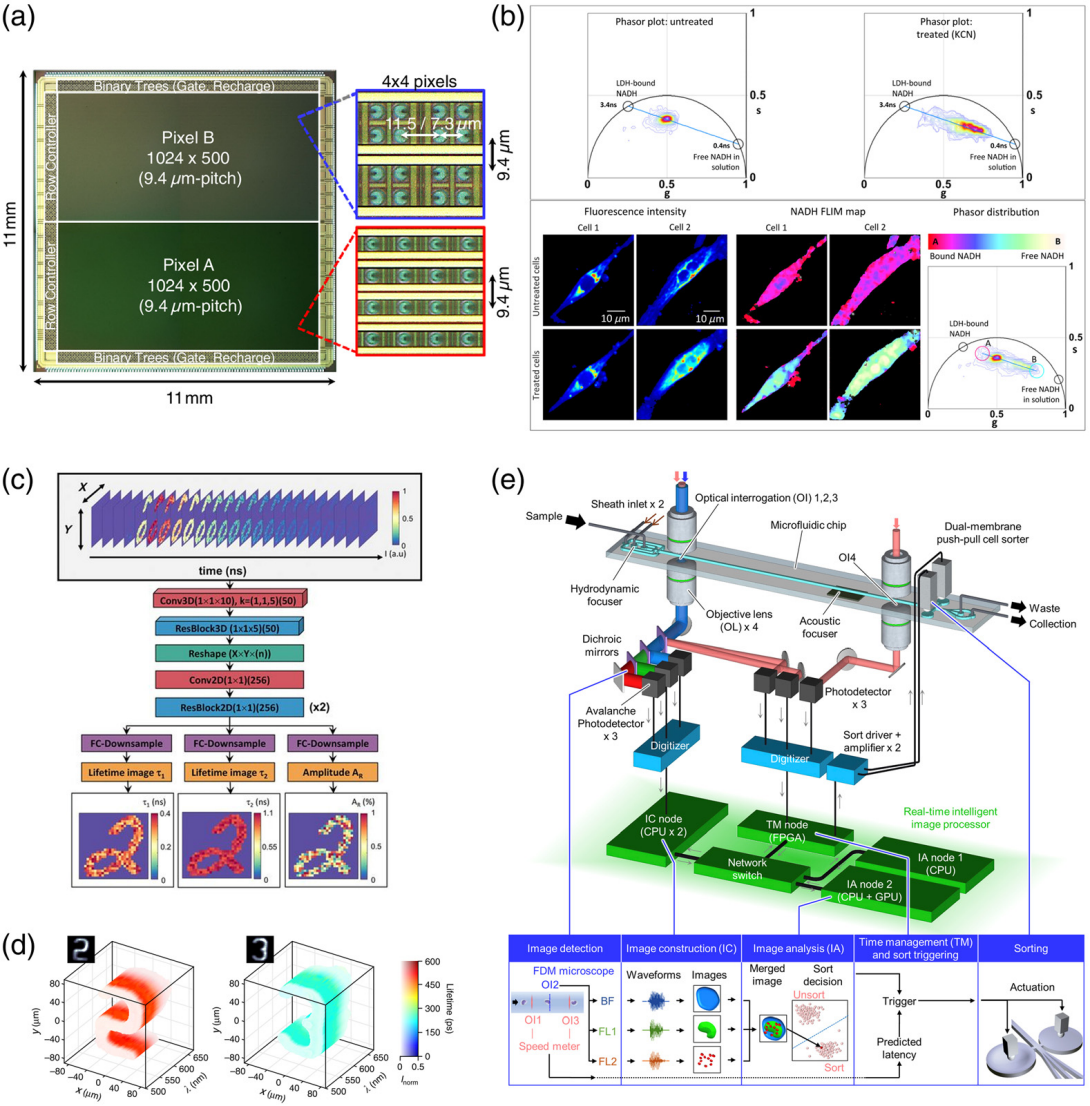
Future prospects of high-speed FLIM. (a) Picture of a SPAD image sensor. The differences between Pixels A and B lie in the number of transistors per pixel and the use of readout transistor sharing. Adapted from Ref. 104, under the Optica Publishing Group OAPA. (b) Example of phasor analysis. The phasor plots show NADH fluorescence lifetimes of human iPS-derived cardiomyocytes without (upper left) and with 4-mM KCN treatment (upper right). The bottom panel displays the fluorescence intensity images, phasor-colored images, and phasor plot of the two representative cells. g=M cos θ: s=M sin θ, where M and θ are the modulation depth reduction and phase shift of fluorescence in the frequency domain, respectively. Adapted from Ref. 105, with permission from Optica. (c) Deep neural network architecture for real-time analysis of fluorescence lifetimes within an entire FOV. Adapted from Ref. 106, with permission from PNAS. (d) Demonstration of high-speed spectrally resolved FLIM of Rhodamine 6G in methanol at 22 mM (left) and 40 mM (right). Adapted from Ref. 107, licensed under CC BY 4.0. (e) Schematic of intelligent image-activated cell sorting. Reproduced from Ref. 76 with permission from Elsevier.

The recent development of SPAD arrays holds significant potential for enhancing the imaging speed of TCSPC.^101^ Unlike conventional image sensors, SPAD arrays with time-tagging mechanisms can enable simultaneous single-photon detection across multiple channels in the entire 2D FOV, thereby accelerating fluorescence decay curve acquisition by accumulating photons in the time domain.^102^ As a result, this parallel detection approach improves imaging speed proportionally to the number of SPAD pixels compared with conventional TCSPC.^103^ Recent advancements have led to the development of SPAD image sensors approaching or exceeding 1 megapixel^104^ [Fig. 5(a)], with some already commercialized, further expanding the possibilities for high-speed TCSPC.^108^ For example, the FLIMera system^109^ from HORIBA Inc. enables wide-field TCSPC-based FLIM with 192×128  pixels at up to 30 frames per second, according to the manufacturer,^110^ and has been recently used for imaging of NADH.^111^ It should be noted that the implementation of many SPAD arrays with a large number of pixels in wide-field TCSPC remains limited due to on-chip circuit complexity, whereas they have been applied to time-gated FLIM.^112^^–^^114^ The integration of these SPAD arrays with more efficient on-chip processing technologies is expected to enable more compact systems for real-time FLIM, broadening the applicability of FLIM in biomedical research and clinical settings, including intraoperative use.^62^^,^^102^^,^^115^^,^^116^

### 4.2 Fit-free Analysis for Fast Fluorescence Lifetime Calculation

Traditional fluorescence lifetime measurements often require fitting fluorescence decay curves to multi-exponential models, which is computationally intensive and challenging when analyzing entire FLIM images. This approach has been accepted due to the historically slow imaging speed of FLIM and the reliance on offline data analysis. However, for high-speed and real-time applications, such as intelligent image-activated cell sorting (refer to Sec. 4.5), computationally efficient techniques for FLIM image analysis are essential.

Incorporating fit-free analysis for FLIM image calculation and interpretation is a promising strategy to accelerate data processing and simplify interpretation. The phasor approach provides a rapid, intuitive, and model-free technique for visualizing fluorescence lifetime distributions in a two-dimensional phase space^117^ [Fig. 5(b)]. In this space, which is designed such that fluorescence lifetime components exhibiting single-exponential fluorescence decays lie on a universal semicircle, fluorophores with different fluorescence lifetimes are plotted at distinct positions. This approach provides insights into the relative contributions of different lifetime components, as the fluorophores with multi-exponential decays are represented by phasor positions corresponding to their intensity-weighted composition.^118^ In the example shown in Fig. 5(b), phasor plots of NADH within cardiomyocytes shift between two distinct states upon potassium cyanide (KCN) treatment, reflecting the redox ratio, NADH/NAD+.^105^ In addition, color mapping based on phasor positions can often offer a more informative visualization of cell states than simple lifetime colormaps. Moreover, both time-domain and frequency-domain FLIM techniques can utilize this approach. The phasor analysis is particularly well-suited for high-speed FLIM, as it enables rapid data visualization and facilitates the identification of heterogeneous fluorophore populations.^119^ Furthermore, deep neural networks and deep learning techniques further enhance the computational speed and accuracy of FLIM analysis, broadening its applicability to real-time imaging^106^ [Fig. 5(c)].

### 4.3 High-speed 3D FLIM

A 3D FLIM, although powerful in providing accurate visualization of complex cellular structures and dynamic biological processes, is inherently time-consuming. This is due to the need for complex 3D fluorescence excitation (e.g., 3D scanning in confocal microscopy), further complicated by the already time-consuming nature of FLIM, which increases the total acquisition time and hinders video-rate 3D imaging.

One promising approach for high-speed 3D imaging is light-field microscopy, which enables single-shot 3D imaging of the target object.^120^ This technique employs a microlens array at the image plane, replacing the conventional image sensor position, to capture angular information of light rays in a single 2D image. By applying appropriate image processing, 3D information can be reconstructed. However, the resolution of the reconstructed 3D image is fundamentally constrained by the number of pixels in the 2D image sensor. Given the limited pixel count of current ultrafast image sensors, light-field imaging has not yet been widely adopted in FLIM. Recently, Ma et al. demonstrated light-field-based computational 3D FLIM using a linear SPAD array.^121^ Their approach captures the 3D scene in a compressed manner using a 1D line sensor, thereby increasing imaging throughput. Although this approach offers a promising pathway toward high-speed 3D FLIM, its volumetric frame rate remains limited to approximately 0.1  frames/sec. Nevertheless, recent advances in light-field microscopy have enabled high-throughput 3D fluorescence imaging flow cytometry,^122^ demonstrating its potential for high-speed biomedical applications. Building on these developments, further improvements in time-resolving imaging architecture and image reconstruction algorithms could pave the way for practical high-speed applications, including high-throughput 3D FLIM flow cytometry.

Light-sheet microscopy can be another important approach for realizing high-speed 3D FLIM. It provides true axial resolution^123^ and causes less phototoxicity and photobleaching than confocal fluorescence microscopy, owing to its thin excitation light sheet that is irradiated orthogonally to the fluorescence collection objective lens.^124^ In this scheme, volumetric imaging can be achieved simply by adding one-axis scanning to 2D wide-field FLIM. Wide-field FLIM in this context has been explored using several techniques: wide-field TCSPC with a position-sensitive microchannel plate-based detector,^125^^,^^126^ time-gating with a commercial SPAD array (SPAD512^2^ from Pi Imaging Technology Inc.),^127^ and time-gating with a Pockels cell,^128^ discussed in Sec. 2.1.^27^^,^^40^^,^^41^ Although light-field microscopy often requires complex computation with intensive computational resources^129^—making it less accessible for non-expert users—and tends to suffer from low spatial resolution and reconstruction artifacts,^130^ light-sheet–based 3D imaging is relatively simple and direct to implement. It offers high spatial resolution, which can even be extended to super-resolution microscopy,^131^ and supports straightforward 3D image reconstruction.

### 4.4 Spectrally Resolved FLIM

Spectrally resolving fluorescence signals, in addition to temporally resolving them, represents another important direction in the advancement of high-speed FLIM. Fluorescence wavelength is widely utilized in biological applications to separate different fluorophores. In addition, finer spectral resolution, achieved with optical gratings, allows for the precise separation of overlapping emission spectra, improving the ability to identify and quantify multiple fluorophores in complex biological samples.^20^^,^^132^ However, spectrally resolved FLIM is typically restricted to slower acquisition speeds, limiting its application to static samples (e.g., fixed cells). This limitation mainly arises from the tradeoff between the number of spectral channels and the number of fluorescence photons per channel, as well as the increased complexity in temporally resolving signals across multiple spectral channels in both hardware and computational processing.

A notable demonstration of high-speed, spectrally resolved FLIM was achieved by Wang et al. using a diffraction grating and a streak camera, enabling spectro-temporal compression of fluorescence images with a temporal resolution of 2 ps, reconstructed by a CS algorithm.^107^ Although this approach represents a significant proof-of-concept, it has so far been validated only for single static fluorescence lifetime image acquisition using a high-concentration Rhodamine 6G solution (tens of millimolar) [Fig. 5(d)]. Future studies are expected to demonstrate its applicability to biological samples and extend its use to time-lapse imaging of dynamic processes. In addition, recent advancements in sensitive detectors, high-efficiency image sensors, and photon collection enhancement technologies are expected to mitigate the limitations inherent to spectrally resolved FLIM, paving the way for high-speed spectrally resolved FLIM. The ability to capture rapid spectral and fluorescence lifetime changes in dynamic biological systems remains largely unexplored, presenting an exciting direction for future research.

### 4.5 Intelligent Image-activated Cell Sorting

Intelligent image-activated cell sorting (iIACS)^76^^,^^133^^,^^134^ is an emerging technology that enables high-throughput and accurate cell sorting by making sort-or-unsort decisions based on deep-learning analysis of single-cell images [Fig. 5(e)]. This approach allows for the rapid and precise isolation of target cells from heterogeneous populations, making it applicable to various fields, including cancer research, stem cell biology, and immunology.^76^ Although iIACS has been demonstrated using bright-field and fluorescence intensity, integrating fluorescence lifetime analysis into the decision-making process (i.e., FLIM-activated cell sorting) could facilitate the isolation of functionally distinct cell subpopulations that are otherwise indistinguishable by intensity-based techniques.^32^ Specifically, it would provide an additional layer of specificity, enabling the distinction of cells based on their metabolic states, molecular interactions, or other intracellular microenvironmental conditions, as fluorescence lifetime enables the characterization of cellular states and phenotypes.^118^^,^^135^^,^^136^

## 5 Conclusions

We provided a comprehensive review of recent advances in high-speed FLIM. Although FLIM has advantages over conventional intensity-based fluorescence microscopy in terms of accuracy and robustness, its imaging speed has traditionally been slow, limiting its applications to static imaging. Recent advances in high-speed FLIM have been primarily driven by innovations in fluorescence detection systems for wide-field imaging and fluorescence excitation strategies for beam-scanning imaging. Specifically, time-gating mechanisms using an image intensifier, Pockels cell, streak camera, or phase-sensitive image sensor have played a crucial role in high-speed wide-field FLIM, whereas serial or parallel fluorescence excitation schemes with spectral or radio-frequency encoding have played a key role in high-speed beam-scanning FLIM. As for video-rate FLIM, raster-scanning two-photon excitation FLIM and several commercial platforms are also available. In addition, we discussed the early biomedical applications of high-speed FLIM, focusing on the observation of fast neuronal activity, video-rate intracellular temperature monitoring, and high-throughput fluorescence lifetime imaging flow cytometry. Furthermore, we examined future prospects and outlined key directions for further development, including the utilization of SPAD arrays, fit-free analysis, 3D FLIM, spectrally resolved FLIM, and iIACS based on FLIM. The development of high-speed FLIM holds great promise for future biomedical applications, providing new possibilities for cell biology, neurology, and biophysics.

## Data Availability

There are no new data presented, as this is a review paper.
